# Piezoresistive Sensitivity, Linearity and Resistance Time Drift of Polysilicon Nanofilms with Different Deposition Temperatures

**DOI:** 10.3390/s90201141

**Published:** 2009-02-23

**Authors:** Changzhi Shi, Xiaowei Liu, Rongyan Chuai

**Affiliations:** 1 MEMS Center, Harbin Institute of Technology, Harbin 150001, Heilongjiang Province, P.R. China E-mail: lxw@hit.edu.cn; 2 Key Laboratory of Micro-Systems and Micro-Structures Manufacturing, Ministry of Education, P.R. China; 3 Information Science and Engineering School, Shenyang University of Technology, Shenyang, Liaoning Province, P.R. China; E-mail: me_sut@163.com

**Keywords:** Polysilicon nanofilm, Piezoresistive effect, Linearity, Resistance time drift, Tunneling, Interstitial-vacancy pair, Deposition temperature

## Abstract

Our previous research work indicated that highly boron doped polysilicon nanofilms (≤100 nm in thickness) have higher gauge factor (the maximum is ∼34 for 80 nm-thick films) and better temperature stability than common polysilicon films (≥ 200nm in thickness) at the same doping levels. Therefore, in order to further analyze the influence of deposition temperature on the film structure and piezoresistance performance, the piezoresistive sensitivity, piezoresistive linearity (PRL) and resistance time drift (RTD) of 80 nm-thick highly boron doped polysilicon nanofilms (PSNFs) with different deposition temperatures were studied here. The tunneling piezoresistive model was established to explain the relationship between the measured gauge factors (GFs) and deposition temperature. It was seen that the piezoresistance coefficient (PRC) of composite grain boundaries is higher than that of grains and the magnitude of GF is dependent on the resistivity of grain boundary (GB) barriers and the weight of the resistivity of composite GBs in the film resistivity. In the investigations on PRL and RTD, the interstitial-vacancy (IV) model was established to model GBs as the accumulation of IV pairs. And the recrystallization of metastable IV pairs caused by material deformation or current excitation is considered as the prime reason for piezoresistive nonlinearity (PRNL) and RTD. Finally, the optimal deposition temperature for the improvement of film performance and reliability is about 620 °C and the high temperature annealing is not very effective in improving the piezoresistive performance of PSNFs deposited at lower temperatures.

## 1. Introduction

For semiconductor materials, the piezoresistive effect was discovered firstly in Ge and Si by Smith in 1954 [1]. In general, the GF in silicon is ∼100 and varies with doping concentration, stress direction and crystal orientation. Noticeably, He *et al.* and Rowe reported that Si nanowires [2, 3] and Al-Si hybrid structures [4] present giant piezoresistances. Although these homogeneous silicon based materials or structures have large piezoresistive responses, there are still several problems, such as p-n junction isolation, high temperature instability, etc., influencing their practical applications. The SOI technology can be brought to solve the isolation problem of devices and substrates, but increases the fabrication cost greatly. The discovery of piezoresistive effect in polysilicon in the 1970s [5] facilitates its applications for sensing devices [6, 7]. As for polysilicon, the p-n junction isolation is avoided, so that the devices can work at higher temperatures. Moreover, polysilicon based devices have the advantages of low cost, facile processing and good thermal stability, compared to homogeneous silicon based devices. Thus, the piezoresistive properties and electromechanical sensors based on the material have been investigated successively for over 20 years [8-11]. Many efforts have been spent on optimizing the film structure by improving fabrication technologies. The most popular technology is chemical vapor deposition, including APCVD, LPCVD, PECVD, etc. Subsequently, by the metal-induced lateral crystallization (MILC) technique, the GF was enhanced to be 60 [12]. However, the sensor chips based on MILC technique could suffer the contamination from the metal layer. LPCVD is a stable and mature CVD method with advantages of low cost, good product uniformity, IC process compatibility, etc., so the PSNFs studied here were prepared by LPCVD to ensure the performance and uniformity of samples.

With the above review, it can be seen that it is necessary to investigate the piezoresistive properties of polysilicon and built up the theoretical model. The experimental results reported by other researchers indicated that the GF of polysilicon common films (PSCFs, film thickness ≥ 200 nm) reaches the maximum as the doping concentration is at the level of 10^19^ cm^-3^, and then decreases drastically as doping concentrations are increased further [9, 13-15]. Based on this phenomenon, the existing piezoresistive theories of polysilicon were established during 1980s∼1990s and used to predict the process steps for the optimization of device performance. In the early models proposed by Mikoshiba [16], Erskine [17] and Germer [18], the contribution of GBs to piezoresistive effect was neglected, thereby resulting in the discrepancy between experimental data and theoretical results at low doping levels. To tackle this issue, Schubert *et al.* took the piezoresistive effect of depletion region barriers (DRBs) arising from carrier trapping at GBs into account and established a theoretical model for calculating GFs [14]. Thereafter, French *et al.* suggested that the piezoresistive effect of p-type polysilicon is not only due to the shift in heavy and light hole band minima relative to each other, but also due to the warpage of two sub-bands [15]. Moreover, the barrier effect of GBs was introduced into the model, achieving the good agreement with the experimental data. Noticeably, it was considered in these models that the PRCs of GBs and DRBs are much lower than that of grain neutral regions. Based on this viewpoint, since the PRC of grain neutral regions (bulk Si) falls off rapidly at high doping concentrations [19], it has been considered that the GF of polysilicon could be degraded sharply with increasing doping concentrations. Accordingly, the optimization of fabrication technologies was emphasized on improving crystallinity and controlling doping concentration to prepare the films with larger grain sizes and lower trap densities. It results in that the research works have been mainly focused on PSCFs and scarcely involving PSNFs.

However, our previous experimental results indicated that boron-doped PSNFs exhibit higher piezoresistive sensitivity (GF ≥ 30) at high doping concentrations than PSCFs (GF is only 20∼25) at the same doping levels [20, 21]. Interestingly, as the doping level exceeds 2×10^20^ cm^-3^, the GF of PSNFs increases with elevating doping concentration [22]. This could not be explained reasonably by the existing piezoresistive model of polysilicon. Additionally, by adjusting doping concentration, PSNFs present good temperature stability (the temperature coefficient of resistance (TCR) is less than 10^-4^/°C, one order of magnitude lower than that of PSCFs; the temperature coefficient of GF (TCGF) is less than 10^-3^/°C, at least twice lower than that of PSCFs) [23, 24]. Moreover, since the signs of TCR and TCGF in PSNFs could be contrary by adjusting technological parameters, the temperature self-compensation of sensors may be achieved. These unique physical properties of heavily doped PSNFs make the material potential for the development of low cost, high temperature stability and miniature volume piezoresistive sensors. Consequently, in order to analyze the piezoresistive properties of highly doped PSNFs, the tunneling effect was introduced and considered as the dominant transport mechanism of carriers traversing GBs in our previous work [20, 22]. The theoretical prediction of GF versus doping concentration gives better agreement with the experimental data than the existing models [21]. Significantly, the research work by He *et al.* showed that silicon nanowires possess giant PRCs [2]. The interpretation of this phenomenon was given by Rowe, and the origin of the giant piezoresistance was considered to be the stress-induced modulation of the surface DRB width [3]. It seems to be relevant to the enhanced GF of PSNFs. However, for highly doped PSNFs here, the DRB width is reduced greatly so that the contribution of DRBs could be neglected and the tunneling effect becomes dominant. It will be demonstrated further in the model calculation later.

In our previous research work, the dependence of the GF on film thickness indicated that highly doped PSNFs with the thickness of ∼80 nm present the highest GF of 34 and the lowest TCR and TCGF. Therefore, the film thickness was selected to be 80 nm in this paper. Nevertheless, the nano-scale thickness is not the direct origin of the enhanced GF. In fact, the reduction of film thickness causes the contraction of grain sizes, which could increase the proportion of GB barriers to grain neutral regions and enhance the influence of the tunneling effect. Namely, the film microstructure (including grain size, GB width, trap density, etc.) has a decisive role in the piezoresistive properties of PSNFs. Deposition temperature is one of the significant technological parameters determining the film structure. Hence, in this paper, the piezoresistive sensitivity of heavily doped PSNFs with different deposition temperatures was firstly investigated. By calculating the tunneling current and the stress-induced changes in the effective mass and the concentration of heavy and light holes, the numerical relationship between the PRCs of GBs and grain neutral regions was obtained. Then the tunneling piezoresistive model of PSNFs was established preliminarily. Because the influences of deposition temperature and heat treatment on film microstructure are quite complicated, the dependence of GF on deposition temperature was analyzed qualitatively based on the obtained theoretical results. On the other hand, for the applications of sensing devices, the reliability and stability of PSNF material are of particular importance. The PRL and RTD of PNSF piezoresistors are two significant parameters characterizing the reliability and stability. Thus, the dependences of PRL and RTD on deposition temperature were given and studied. And the IV model was established to describe GBs and analyze PRL and RTD qualitatively. Also, the influence of residual H atoms in polysilicon was taken into account for RTD.

## 2. Experimental Details

### 2.1. Film preparation

Before preparing films, a 1 μm-thick SiO_2_ layer was grown on the 510 μm-thick (111) Si wafers (4 inch diameter) by thermal oxidization at 1100°C. Then, the 80 nm-thick PSNFs were deposited on the thermally oxidized Si substrates by LPCVD at a pressure of 45∼55 Pa over a temperature range of 560∼670°C. The reactant gas was SiH_4_ and the flow rate was 50 mL/min. Since the films deposited at 560∼600°C exhibited amorphous appearance mixed with polycrystals, the pre-annealing was performed on them in dry N_2_ at 950°C for 30 min to induce the recrystallization of amorphous regions.

It has been reported elsewhere [15] that the GF is higher in boron doped polysilicon than in phosphorus doped polysilicon with the same fabrication process. Therefore, only the boron doped PSNFs were investigated here. For the dopant implantation, boron ions were implanted into the samples at a dose of 2×10^15^ cm^-2^ at 20 keV. For the sake of dopant activation and ion implantation damage elimination, the post-implantation annealing was carried out in N_2_ atmosphere at 1080°C for 30 min. Then, the doping concentration was estimated to be 2×10^20^ cm^-3^.

### 2.2. Film microstructure characterization

**Figure 1.**
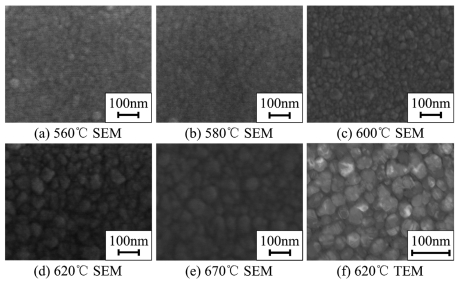
SEM and TEM images of PSNFs deposited at different temperatures.

The surface morphology of PSNFs was characterized by SEM, as shown in Figures 1(a)-(e). It can be seen that the grain size increases with elevating deposition temperature. This indicates that the crystallinity of PSNFs can be improved by raising deposition temperature. The grain size can be determined by TEM, as shown in Figure 1(f). The mean grain size of 620°C samples is estimated to be 40 nm approximately. With the deposition temperature varying from 560°C to 670°C, the mean grain size increases from 30 nm to 70 nm. For the sake of clarity, the 560∼600°C films undergoing the pre-annealing of 950°C are called recrystallized (RC) PSNFs, while the 620∼670°C films are called directly crystallized (DC) PSNFs. From Figure 1, it can be seen that the borders between GBs and grains of RC PSNFs are obscure as well as the 670°C samples. It shows that the GBs of the above-mentioned samples contain a large number of amorphous phases.

**Figure 2.**
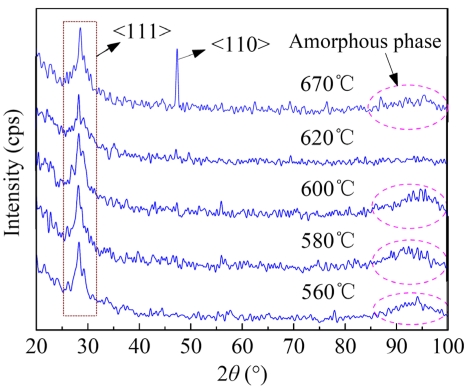
XRD spectra of PSNF samples deposited at different temperatures.

In order to analyze the film microstructure, the XRD experiment was performed on the samples. In the XRD spectra shown in Figure 2, all the <111> peaks are attributed to Si substrates. The clear <110> peak of 670°C PSNFs is due to the preferred grain growth along <110> orientation, while the other PSNFs are oriented randomly. Furthermore, it should be noted that the broad peaks (2*θ*=85∼100°) related to amorphous phases appear on the spectra of RC and 670°C PSNFs, thereby testifying the existence of amorphous phases at GBs. Because amorphous phases in the 620°C PSNFs are much fewer, no remarkable broad peak is observed. The peak intensity and FWHM of RC PSNFs are larger than those of the 670°C ones. It demonstrates that the crystallinity of RC PSNFs is lower than DC ones. The broad peak of 670°C samples is likely due to the preferred growth aggravating disordered states of GBs.

### 2.3. Cantilever beam fabrication

**Figure 3.**
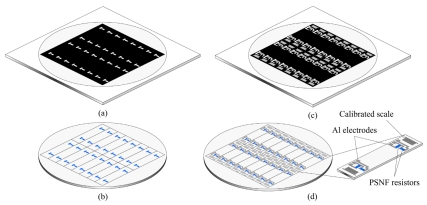
Schematic diagram of mask plates and sample wafers in the fabrication of cantilever beams. (a) The mask plate for patterning resistors. (b) The sample wafer after patterning resistors. (c) The mask plate for patterning electrodes and calibrated scales. (d) The sample wafer and the cantilever beam after fabricating electrodes and calibrated scales.

For measuring GFs, the cantilever beams were fabricated based on photolithography and etching technologies. Firstly, the sample wafers were ultrasonically degreased with methylbenzene, acetone and ethanol for 5 min in each and then rinsed repeatedly in de-ionized water. The cleaned samples were pre-baked at 120°C for 15 min. Next, after spin-coating with positive photoresist and a soft-bake at 90°C for 10 min, the samples were exposed for 90 s using the mask plate as shown in Figure 3(a) and developed in the 5% NaOH solution. Then, a hard-bake for 25 min was performed at 120°C for the successive etching process. After photolithography, the samples were etched in HNO_3_/HAc/HF (4:1:1) solution to form PSNF resistors and then rinsed in de-ionized water. The photoresist was removed by acetone to obtain the sample wafers with PSNF resistors as shown in Figure 3(b).

Before depositing metal, the samples were dipped in HF/H_2_O (1:10) for 1∼2 s to remove the native oxide. The Al layer was evaporated onto the samples by vacuum evaporation. Then, the positive photo-resist was coated and patterned in the same process as the resistor fabrication. The schematic diagram of mask plate is shown in Figure 3(c). The Al layer was etched in concentrated phosphorous acid at 80∼100°C to form electrodes. The electrode fabrication was completed by removing the photoresist left.

**Figure 4.**
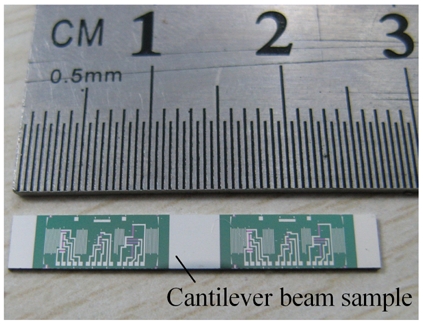
Photograph of an actual cantilever beam sample.

After scribing, the sample wafers were divided into individual cantilever beams of 26 mm×4 mm, as shown in Figure 3(d). Then, the samples were alloyed at 420°C for 5 min in N_2_ to form ohmic contact. Finally, on the actual cantilever beam sample given in Figure 4, two groups of PSNF piezoresistors were fabricated. Each group consists of three sets of longitudinal and transversal piezoresistors with length-width ratios of 1:4, 2:1 and 8:1, respectively. And the current directions through longitudinal resistors were aligned with the <110> orientation. Also, the Al calibrated scales were fabricated near both ends of cantilever beams for measuring the arm of applied force.

### 2.4. Measurement of gauge factor

**Figure 5.**
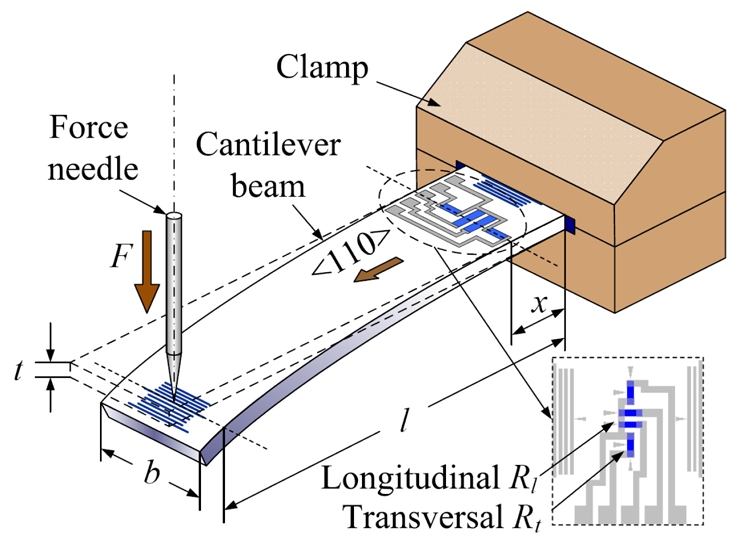
Schematic diagram of test setup for measuring gauge factor.

The GF test setup is shown in Figure 5. Either end of the cantilever beam is fixed by the clamp. The piezoresistors are connected to the electric instruments through Al electrodes.

When an axial compressive force *F* caused by the force needle and loading weights is applied to the free end of the cantilever beam, the arm of force *l* and the location of the measured piezoresistors *x* can be measured with the calibrated scales, using an optical microscope. And the strain *ε*(*x*) at site *x* is expressed as:

(1) $$\varepsilon (x)=\frac{6(l-x)\cdot F}{bt^{2}Y},$$

where *b* and *t* are the width and the thickness of the cantilever beam (*b*, *t* ≪ *l* here), respectively. *Y* is Young's modulus. By measuring the initial resistance value *R*_0_ (without strain) and the resistance value *R* under the strain *ε* by a Keithley 2000 digital multimeter, the GF can be calculated by:

(2) $$\text{GF}=\frac{R-R_{0}}{R_{0}\cdot \varepsilon }=\frac{\Delta R}{R_{0}\cdot \varepsilon }$$

### 2.5. Measurement of resistivity and resistance time drift

**Figure 6.**
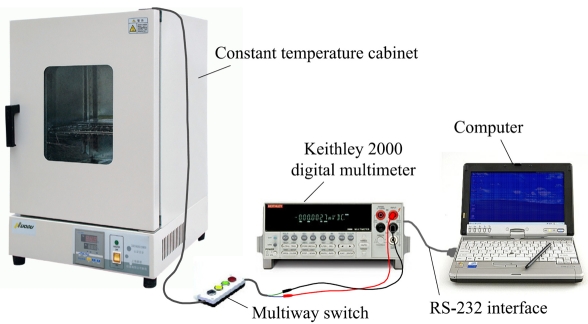
Test system of RTD properties.

By measuring the I-V characteristics of PSNF resistors with different length-width ratios, the film resistivity *ρ* can be calculated from the average measured resistance value *R*_ave_:

(3) $$\rho =\frac{d\cdot W\cdot R_{\text{ave}}}{L}=\frac{W}{L}R_{S},$$

where *L, W, d* and *R_S_* are the length, width, thickness and sheet resistance of PSNF resistors, respectively. The RTD is defined as the relative change in the resistance value per unit time and expressed by *RTD* = (*resistance drift*/*initial resistance*)/*testing time*. For measuring RTD, the samples were placed in a constant temperature cabinet and the resistances were monitored by the Keithley 2000 digital multimeter for 24 h at 23°C (room temperature). And the measured data were collected through the RS-232 interface to the computer. The test system of RTD properties is shown in Figure 6.

## 3. Piezoresistive sensitivity and tunneling piezoresistive theory

In this section, based on the tunneling piezoresistive effect, the tunneling current of carriers traversing GBs is derived, and the relationship between the PRCs of GBs and grain neutral regions is presented. Then, the measured results of resistivity and GF are given. Finally, the dependences of the GFs on deposition temperature is analyzed and discussed based on the tunneling piezoresistive theory.

### 3.1. Carrier transport mechanisms through grain boundaries

Polysilicon can be considered as composed of small crystals joined together by GBs. Each crystal is viewed as a Si single crystal, while the GBs are full of defects and dangling bonds and form extremely thin amorphous layers. The forbidden band width of GBs is larger than that of monocrystalline silicon (1.12eV [25, 26]) and approaches that of amorphous silicon (1.5-1.6eV [27]). The Fermi level is pinned near the midgap at GBs. In this case, the GB barriers are formed to hinder carriers from traversing GBs. Moreover, dangling bonds at GBs can be occupied by carriers and dopant atoms, so the DRBs are created on the sides of GBs. As a result, the GB barriers and the DRBs form the composite GB barriers.

**Figure 7.**
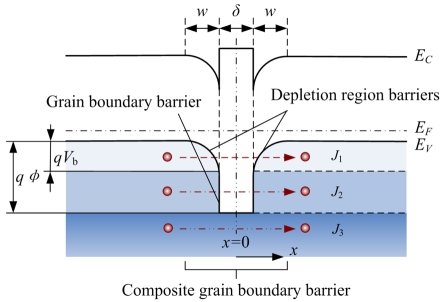
Energy band structure and carrier transport mechanisms near grain boundaries.

Theoretically, carriers pass through GBs by two transport mechanisms of thermionic emission and tunneling. For simplification, the carrier transport is considered to be one-dimensional. So, according to the kinetic energy *E_x_* of carriers, there are three current components in the conduction current of carriers traversing GBs (Figure 7), where *w, δ, qϕ* and *qV_b_* are the DRB width, the GB width, the GB barrier height and the DRB height, respectively. At very low temperatures, *E_x_*<*qV_b_*, carriers traverse the composite GBs only by tunneling, forming the field emission current *J*_1_; At intermediate temperatures, *qV_b_*<*E_x_*< *qϕ*, carriers cross the DRBs by thermionic emission and penetrate the GB barrier by tunneling, forming the composite current *J*_2_; At very high temperatures, *E*_x_> *qϕ*, carriers traverse the composite GB completely by thermionic emission, forming the thermionic emission current *J*_3_. In the temperature range of polysilicon devices working, *J*_2_ is dominant, and *J*_1_ and *J*_3_ could be neglected [25].

In our tunneling piezoresistive model, the piezoresistive effect of GBs is due to that the stress-induced deformation gives rise to the split-off of the degenerate heavy and light hole sub-bands, thereby causing the carrier transfer between two bands and the conduction mass shift. Inside each grain, due to the single crystal nature of grain neutral regions, the GF of this regions, *GF*_g_, is dependent on the PRC of Si single crystals, *π_g_*. The GF of composite GBs, *GF*_b_, is dependent on the PRC of DRBs (*π_d_*) and the PRC of GB barriers (*π_δ_*). Hence, in order to explain the piezoresistive behavior of PSNFs with different deposition temperatures theoretically, it is necessary to deduce the relationship between *π_g_*, *π_d_* and *π_δ_*.

For DRBs, based on the dependence of thermionic emission current on strain, the relational expressions of longitudinal PRC *π_dl_* and transversal PRC *π_dt_* in the [111] orientation have been derived in our previous work [28] and expressed as:

(4) $$\pi_{\text{dl}}=0.525\pi_{\text{gl}},$$

(5) $$\pi_{\text{dt}}=0.616\pi_{\text{gt}},$$

where *π_gl_* and *π_gt_* are the longitudinal and transversal PRCs of p-type monocrystalline silicon in the [111] orientation, respectively.

### 3.2. Tunneling current through grain boundary barriers

**Figure 8.**
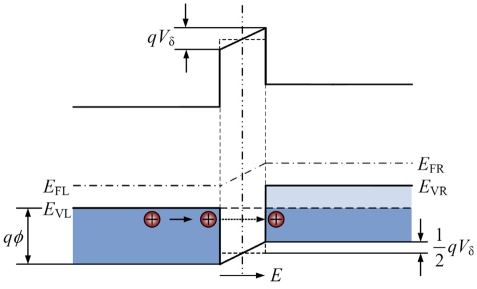
Energy band diagram and tunneling mechanism of GB barrier omitting DRBs.

Before deducing the PRC *π_δ_*, the conduction current of carriers penetrating GB barriers must be determined. Figure 8 provides the energy band diagram and tunneling mechanism of GB barrier omitting DRBs. It is assumed that the voltage drop over the GB barrier is *V_δ_*. Using Fermi-Dirac statistics, the number of holes having energy within the range d*E_x_* incident from left to right on the GB barrier per unit time per unit area is [29]:

(6) $$N(T,\xi ,E_{x})dE_{x}=\frac{4\pi \cdot m_{d}kT}{h^{3}}\ln\left\{1+\exp\left[\frac{-\left(E_{x}+\xi \right)}{kT}\right]\right\}dE_{x},$$

where *m_d_* is the effective mass of holes for state density, *ξ* = *E_F_ - E_V_*, is the difference of Fermi level and valence band edge, *h* is Planck's constant, *k* is Boltzmann's constant, *T* is the absolute temperature.

The GB width *δ* is very small (around 1nm), and the number of the holes with high energies around *qϕ* is few. Hence, when calculating the current density, the oblique distribution of energy band at the top of GB barrier in Figure 8 can be substituted by the horizontal line approximately. So, the probability of carriers with the energy *E_x_* (0 ≤ *E_x_* ≤ *qϕ* − *qVδ*/2) tunneling the GB barrier is given by:

(7) $$D(E_{x})=\exp\left\{\frac{-4\pi \delta }{h}{\left[2m_{i}(a-E_{x})\right]}^{\overset{1}{\;}/\underset{2}{\;}}\right\},$$

(8) $$a=q\phi -\frac{1}{2}qV_{\delta },$$

where *m_i_* is the hole effective mass in the tunneling direction. In Figure 8, the left valence band edge *E_VL_* is taken to be the zero point of energy. By deducing from Eqs. (6)-(8), the current density of holes tunneling GB barrier from left to right is:

(9) $$S_{\text{LR}}=\int_{0}^{a}N(T,\xi ,E_{x})\cdot D(E_{x})dE_{x}.$$

The current density of holes tunneling GB barrier from right to left is:

(10) $$S_{\text{RL}}=\int_{0}^{a}N(T,\xi^{\prime },E_{x})\cdot D(E_{x})dE_{x},$$

(11) $$\xi^{\prime }=\xi +qV_{\delta }.$$

By simplifying the logarithmic term in Eq. (6) into the exponential form, Eq. (9) can be expressed as:

(12) $$S_{\text{LR}}=\frac{4\pi \cdot m_{d}kT}{h^{3}}\int_{0}^{a}\exp\left[\frac{-(E_{x}+\xi )}{kT}\right]\cdot D(E_{x})dE_{x}.$$

Considering the fact that the holes gather mostly near the valence band edge, when solving the integration in Eq. (12), the square root term is expended by the Taylor's series as follows:

(13) $${\left(a-E_{x}\right)}^{1/2}=\sqrt{a}-\frac{E_{x}}{2\sqrt{a}}+\cdots .$$

Substituting Eq. (13) into Eq. (7), Eq. (12) can be solved out by integrating:

(14) $$S_{\text{LR}}=\frac{4\pi m_{d}k^{2}T^{2}}{h^{3}c_{1}}[\exp\left(-\frac{2\pi \delta }{h}\sqrt{2m_{i}a}-\frac{a+\xi }{kT}\right)-\exp\left(-\frac{4\pi \delta }{h}\sqrt{2m_{i}a}-\frac{\xi }{kT}\right)],$$

where

(15) $$c_{1}=\frac{2\pi \delta }{h}kT\sqrt{\frac{2m_{i}}{a}}-1.$$

Similarly,

(16) $$S_{\text{RL}}=\frac{4\pi m_{d}k^{2}T^{2}}{h^{3}c_{1}}[\exp\left(-\frac{2\pi \delta }{h}\sqrt{2m_{i}a}-\frac{a+\xi^{\prime }}{kT}\right)-\exp\left(-\frac{4\pi \delta }{h}\sqrt{2m_{i}a}-\frac{\xi^{\prime }}{kT}\right)].$$

Then, the current density of tunneling the GB barrier can be given by:

(17) $$\begin{array}{ll} J_{\delta } & =q(S_{LR}-S_{RL})\\ & =q\frac{4\pi m_{d}k^{2}T^{2}}{h^{3}c_{1}}\exp\left(-\frac{\xi }{kT}\right)\cdot [\exp\left(c_{2}-\frac{a}{kT}\right)-\exp\left(c_{2}-\frac{a+qV_{\delta }}{kT}\right)-\exp\left(2c_{2}\right)+\exp\left(2c_{2}-\frac{qV_{\delta }}{kT}\right)], \end{array}$$

where

(18) $$c_{2}=-\frac{2\pi \delta }{h}\sqrt{2m_{i}a}.$$

In the case of low voltage bias (*qV_δ_* ≪ *kT*), the exponential terms in Eq. (17) can be expanded by using the Taylor's series. After taking the first order approximation, it yields:

(19) $$J_{\delta }=\frac{4\pi q^{2}m_{d}kT}{h^{3}c_{1}}\exp\left(-\frac{\xi }{kT}\right)\cdot \left[\exp\left(c_{2}-\frac{a}{kT}\right)-\exp\left(2c_{2}\right)\right]\cdot V_{\delta }.$$

Considering the hole concentration formula:

(20) $$p=N_{V}\exp\left(\frac{-\xi }{kT}\right)=2{\left(\frac{2\xi m_{d}kT}{h^{2}}\right)}^{\frac{3}{2}}\exp\left(\frac{E_{V}-E_{F}}{kT}\right),$$

and then Eq. (19) can be rewritten as:

(21) $$J_{\delta }=\frac{pq}{c_{1}}{\left(\frac{kT}{2\pi m_{d}}\right)}^{1/2}\left[\exp\left(c_{2}-\frac{a}{kT}\right)-\exp\left(2c_{2}\right)\right]\cdot \frac{qV_{\delta }}{kT}=pJ_{\delta 0}.$$

When two sub-bands split off under an axial stress, the total tunneling current (*J_δ_*) consists of tunneling currents of heavy holes (*J_δ_*_1_) and light holes (*J_δ_*_2_) and can be expressed as:

(22) $$J_{\delta }=\sum_{j=1}^{2}J_{\delta j}=J_{\delta 1}+J_{\delta 2},$$

(23) $$J_{\delta j}=p_{j}{\left(J_{\delta 0}\right)}_{j},$$

where *J_δj_* is the tunneling current component of degenerate sub-band, *p_j_* is the corresponding hole concentration, the subscript *j*=1, 2, represents the heavy and light hole sub-bands, respectively.

### 3.3. Piezoresistance coefficient of grain boundary barriers

When the heavy and light hole sub-bands split off under stress, the band shift *ε*′ is defined as the shift of two split-off sub-bands (*E*_V1_ and *E*_V2_) relative to the initial degenerate band (*E*_V_). For the sake of simplification, the applied axial stress is assumed to be along the <111> orientation. According to the result of the cyclotron resonance experiment by Hensel and Feher [30], the effective mass of holes under an axial stress is obtained in Table 1, where *m_lj_* and *m_tj_* are the longitudinal and transversal effective mass of holes at the sub-band *E*_Vj_, respectively.

The split-off heavy and light hole sub-bands are *E*_V_+*ε*′ and *E*_V_-*ε*′, respectively. By differentiating Eq. (20) and substituting d*E*_V_ by *ε*′, the concentration changes of two sorts of holes are, respectively:

(24) $$\Delta p_{1}=N_{v1}\exp\left(\frac{E_{V}-E_{F}}{kT}\right)\cdot \frac{\varepsilon^{\prime }}{kT},$$

(25) $$\Delta p_{2}=-N_{v2}\exp\left(\frac{E_{V}-E_{F}}{kT}\right)\cdot \frac{\varepsilon^{\prime }}{kT}.$$

When the uniaxial stress is *σ̄*, the band shift *ε*′ is [30]:

(26) $$\varepsilon \prime =\frac{1}{3}D_{u}{C_{44}}^{-1}\bar{\sigma },$$

where *D_u_* is deformation potential constant, *C*_44_ is the corresponding elastic stiffness constant. Due to the different effective mass of heavy and light holes, the change in the corresponding hole concentra-tions can lead the tunneling current *J_δ_* to vary, which is the principle of tunneling piezoresistive effect. The relative change of the equivalent tunneling resistivity is:

(27) $$\frac{\Delta \rho_{\delta }}{\rho_{\delta }}=-\frac{\Delta J_{\delta }}{J_{\delta }}=-\frac{\Delta p_{1}{\left(J_{\delta 0}\right)}_{1}+\Delta p_{2}{\left(J_{\delta 0}\right)}_{2}}{p_{1}{\left(J_{\delta 0}\right)}_{1}+p_{2}{\left(J_{\delta 0}\right)}_{2}}.$$

Substituting Eqs. (20), (24) and (25) into Eq. (27), it yields:

(28) $$\frac{\Delta \rho_{\delta }}{\rho_{\delta }}=\frac{1-{\left(\frac{m_{d1}}{m_{d2}}\right)}^{3/2}\cdot \frac{{\left(J_{\delta 0}\right)}_{1}}{{\left(J_{\delta 0}\right)}_{2}}}{1+{\left(\frac{m_{d1}}{m_{d2}}\right)}^{3/2}\cdot \frac{{\left(J_{\delta 0}\right)}_{1}}{{\left(J_{\delta 0}\right)}_{2}}}\cdot \frac{\varepsilon^{\prime }}{kT}.$$

From Eqs. (21)-(23), it results in:

(29) $$\frac{{\left(J_{\delta 0}\right)}_{1}}{{\left(J_{\delta 0}\right)}_{2}}=\frac{{\left(c_{1}\right)}_{2}\left\{\exp\left[{\left(c_{2}\right)}_{1}-\frac{a}{kT}\right]-\exp\left[2{\left(c_{2}\right)}_{1}\right]\right\}}{{\left(c_{1}\right)}_{1}\left\{\exp\left[{\left(c_{2}\right)}_{2}-\frac{a}{kT}\right]-\exp\left[2{\left(c_{2}\right)}_{2}\right]\right\}},$$

where (*c*_1_)*_j_* and (*c*_2_)*_j_* can be determined by Eqs. (15) and (18), respectively. According to the experiment data provided by Mandurah [25], the GB width *δ* is set to be 1nm and the GB barrier height *qϕ* is about 0.6eV. Thus, for heavy holes, *j*=1, (*c*_1_)_1_ and (*c*_2_)_1_ are calculated to be -0.84 and -3.72, respectively; for light holes, *j*=2, (*c*_1_)_2_ and (*c*_2_)_2_ are calculated to be -0.94 and -1.46, respectively. In general, *qV_δ_* ≪ *qϕ*, it can be obtained from Eq. (8) that *a* ≈ *qϕ*. From Eq. (29) and Table 1, it yields:

(30) $${\left(\frac{m_{d1}}{m_{d2}}\right)}^{3/2}=\sqrt{\frac{m_{l1}{m_{t1}}^{2}}{m_{l2}{m_{t2}}^{2}}}=1.180,$$

(31) $$\frac{{\left(J_{\delta 0}\right)}_{1}}{{\left(J_{\delta 0}\right)}_{2}}=1.22\times 10^{-2}.$$

Finally, using Eqs. (26), (28)-(31), the longitudinal PRC of GB barriers along the <111> orientation is expressed as:

(32) $$\pi_{\delta l}=\frac{\Delta \rho_{\delta }}{\rho_{\delta }\bar{\sigma }}=\frac{0.972}{3}\cdot D_{u}{C_{44}}^{-1}\cdot \frac{1}{k_{0}T}.$$

Similarly, the transversal PRC along <111> orientation is:

(33) $$\pi_{\delta t}=\frac{\Delta \rho_{\delta }}{\rho_{\delta }\bar{\sigma }}=\frac{-0.684}{3}\cdot D_{u}{C_{44}}^{-1}\cdot \frac{1}{k_{0}T}.$$

From our previous research results, the longitudinal and transversal PRCs of p-type monocrystalline silicon (grain neutral regions) under a uniaxial stress *σ̄* applied along the <111> orientation can be expressed as follows, respectively [28]:

(34) $$\pi_{\text{gl}}=\frac{0.695}{3}\cdot D_{u}{C_{44}}^{-1}\cdot \frac{1}{k_{0}T},$$

(35) $$\pi_{\text{gt}}=-\frac{0.435}{3}\cdot D_{u}{C_{44}}^{-1}\cdot \frac{1}{k_{0}T}.$$

Comparing Eqs. (32) and (33) with (34) and (35) accordingly, it yields:

(36) $$\pi_{\delta t}=1.4\pi_{\text{gl}}.$$

(37) $$\pi_{\delta t}=1.6\pi_{\text{gt}}.$$

From Eqs. (36) and (37), it can be seen that the PRCs *π_δ_* and *π_g_* present a proportional relationship and the PRC *π_δ_* is larger than *π_g_*.

### 3.4. Piezoresistance coefficient of composite grain boundaries

From the above theoretical analysis, it can be seen that both the PRCs of DRBs and GB barriers are proportional to the PRC of grain neutral regions. Noticeably, according to Eqs. (4), (5), (36) and (37), the PRC of DRBs *π_d_* is lower than *π_g_*, while the PRC of GB barriers *π_δ_* is higher than *π_g_*. Therefore, the relationship between the PRC of composite GBs *π_b_* and the PRC of grain neutral regions *π_g_* is dependent on the weights of the equivalent resistivity *ρ_d_* (for DRBs) and *ρ_δ_* (for GB barriers) in the equivalent resistivity *ρ_b_* of composite GBs. In this case, *π_b_* can be expressed as:

(38) $$\pi_{b}=\frac{\Delta \rho_{b}}{\rho_{b}\bar{\sigma }}=\frac{\rho_{d}}{\rho_{b}}\cdot \frac{\Delta \rho_{d}}{\rho_{d}\bar{\sigma }}+\frac{\rho_{\delta }}{\rho_{b}}\cdot \frac{\Delta \rho_{\delta }}{\rho_{\delta }\bar{\sigma }}=\frac{\rho_{d}}{\rho_{b}}\pi_{d}+\frac{\rho_{\delta }}{\rho_{b}}\pi_{\delta },$$

where

(39) $$\rho_{b}=\rho_{d}+\rho_{\delta }.$$

If the potential drops across DRBs on the left and right hand sides of the GB are denoted by *V_L_* and *V_R_*, respectively; then the potential drops on DRBs and GB barrier are *V_L_*+*V_R_* and *V_δ_*, respectively. So,

Eq. (38) can be expressed as follows:

(40) $$\pi_{b}=\frac{V_{L}+V_{R}}{V_{0}}\pi_{d}+\frac{V_{\delta }}{V_{0}}\pi_{\delta },$$

(41) $$V_{0}=V_{\delta }+V_{L}+V_{R}$$

where *V*_0_ is the potential drop over the composite GB. Thus, it can be seen that determining the proportional relationship between *V_L_*+*V_R_* and *V_δ_* is the key to solve out *π_b_*.

Because the polysilicon usually work under low current and low voltage bias, the condition of *V_L_*+*V_R_*<4*V_b_* can be always satisfied. Then, the relationship of *V_L_, V_R_, V_b_* and *V_δ_* can be obtained [25]:

(42) $$2V_{b}^{1/2}={\left(V_{b}+V_{R}\right)}^{1/2}+{\left(V_{b}-V_{L}\right)}^{1/2},$$

(43) $$V_{\delta }=\delta {\left(\frac{qN_{A}}{2\varepsilon_{s}\varepsilon_{0}}\right)}^{1/2}\left[{\left(V_{b}+V_{R}\right)}^{1/2}-{\left(V_{b}-V_{L}\right)}^{1/2}\right].$$

**Figure 9.**
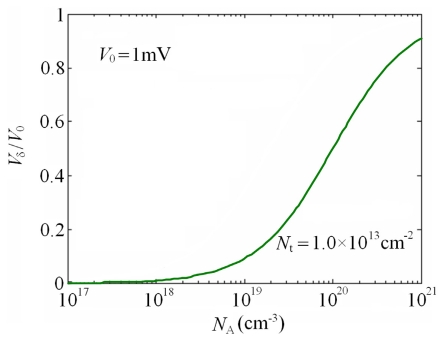
Distribution curve of *V_δ_* normalized to *V*_0_ as a function of *N_A_*. The trap density *N_t_* is taken to be 1.0×10^13^ cm^-2^. The voltage drop *V*_0_ on each composite GB is set to be 1mV.

According to the approximation of DRBs, it yields

(44) $$V_{b}=\frac{qN_{A}w^{2}}{2\varepsilon_{s}\varepsilon_{0}},$$

(45) $$w=\frac{N_{t}}{2N_{A}},$$

where *N_A_* is the boron doping concentration, *N_t_* is the trap density at GBs, *ε_s_* and *ε*_0_ are the relative and vacuum dielectric constants of Si, respectively. In this paper, *N_t_* is taken to be 1.0×10^13^ cm^-2^. By calculating, the distribution of *V_δ_* normalized to *V*_0_ as a function of *N_A_* is provided in Figure 9.

By substituting Eqs. (4), (5), (36) and (37) into Eq. (39), the relational expression of longitudinal PRCs of composite GBs (*π_bl_*) and grain neutral regions (*π_gl_*) is obtained as:

(46) $$\pi_{bl}=0.525\left(1-\frac{V_{\delta }}{V_{0}}\right)\pi_{gl}+1.40\frac{V_{\delta }}{V_{0}}\pi_{gl}$$

For p-type monocrystalline silicon, π_11_+2π_12_ ≪2π_44_, it yields [1]:

(47) $$\pi_{\text{gl}}\approx \frac{2}{3}\pi_{44}$$

**Figure 10.**
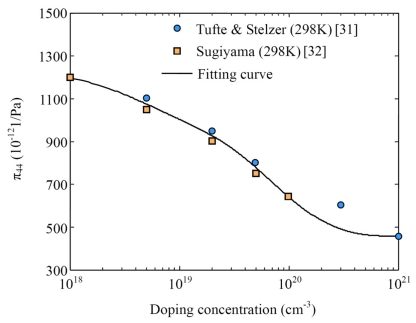
Fitting curve of shear piezoresistance coefficient *π*_44_ on the doping concentration *N_A_* based on the experimental results from Tufte [31] and Sugiyama [32].

**Figure 11.**
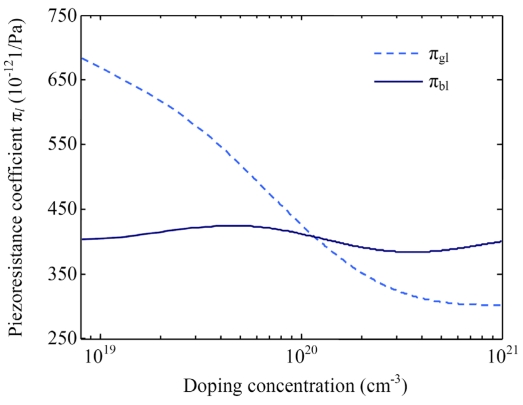
Dependences of longitudinal PRCs *π_bl_* and *π_gl_* on the doping concentration *N_A_*.

According to the experimental results from Tufte [31] and Sugiyama [32], the fitting curve of shear PRC *π*_44_ on *N_A_* is presented in Figure 10. Consequently, using Eqs. (46), (47) and the curves in Figure 9 and 10, the dependences of the longitudinal PRCs *π_bl_* and *π_gl_* on *N_A_* is obtained, as shown in Figure 11. It can be seen that the relationship between the PRC *π_bl_* and *N_A_* is complicated and the variation range of *π_bl_* is smaller than that of *π_gl_*. However, the PRC of composite GBs is larger than that of grain neutral regions at high doping concentrations, which is opposite to the existing models. This conclusion could be used to explain the dependence of GFs on deposition temperature.

### 3.5. Resistivity and gauge factor versus deposition temperature

**Figure 12.**
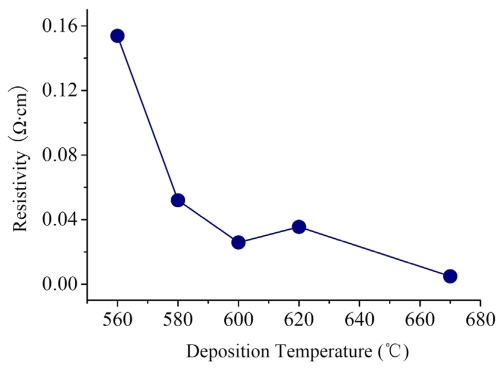
Resistivity of boron doped PSNFs versus deposition temperature.

Figure 12 provides the resistivity versus the deposition temperature. It can be seen that the resistivity changes from 1.54×10^-1^ to 4.9×10^-3^ Ω·cm with elevating deposition temperature. Considering the experiment results that the grain size increases with raising deposition temperature, it indicates that the weight of the resistivity of composite GBs *ρ_b_* in the film resistivity *ρ* is reduced by increasing deposition temperature. Because *ρ_b_* is dependent on the resistivity of GB barriers *ρ_δ_* and the resistivity of DRBs *ρ_d_* (i.e., *ρ_b_* = *ρ_δ_* + *ρ_d_*), the elevation of deposition temperature might reduce either of *ρ_δ_* and *ρ_d_*. According to the SEM and XRD results, there are more amorphous contents in RC PSNFs than in DC PSNFs. The existence of amorphous phases at GBs could increase the resistivity *ρ_δ_*. On the other hand, the high doping concentration narrows the DRB width to a few angstroms, so that the contribution of *ρ_d_* to *ρ_b_* could be neglected. Therefore, at high doping concentration, the resistivity *ρ_b_* is mainly dependent on the resistivity *ρ_δ_*, and the reduction of amorphous contents at GBs caused by elevating deposition temperature is responsible for the falloff of the film resistivity *ρ*. However, the resistivity of 620°C samples is slightly higher than that of 600°C ones. It is likely due to the recrystallization of 600°C samples after the pre-annealing at 950°C, which is discussed in details in the next section.

**Figure 13.**
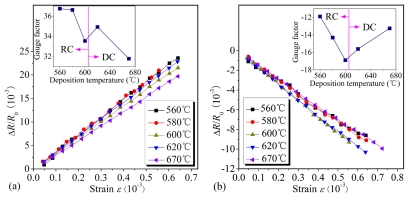
(a) Dependences of the resistance change Δ*R*/*R*_0_ in longitudinal piezoresistors on strain *ε* with different deposition temperatures, and the longitudinal GF vs. deposition temperature. (b) Dependences of Δ*R*/*R*_0_ in transversal piezoresistors on strain *ε* with different deposition temperatures, and the transversal GF vs. deposition temperature.

The dependences of the resistance change Δ*R*/*R*_0_ in longitudinal and transversal piezoresistors on the strain *ε* with different deposition temperatures are shown in Figures 13(a) and (b), respectively. Obviously, the longitudinal and transversal piezoresistances vary linearly with the strain. From the insets of Figure 13(a) and (b), it can be seen that RC PSNFs and DC PSNFs exhibit different piezoresistive properties. And the critical deposition temperature differentiating RC PSNFs and DC PSNFs is around 605°C. The samples deposited below this critical temperature present amorphous appearance mixed with polycrystals, while the samples above this value present better polysilicon appearance. This critical value is consistent with the result by French *et al.* [15].

For the longitudinal piezoresistive sensitivity, it can be seen in Figure 13(a) that the GFs of RC or DC PSNFs decrease with elevating deposition temperature. As discussed above, the amorphous contents at GBs are reduced by raising deposition temperature. For RC PSNFs, when the deposition temperature is lowered, the crystallinity of samples is aggravated and there are more amorphous phases existing at GBs. The increase of amorphous phases raises the resistivity *ρ_δ_*. Moreover, the deficient crystallinity increases the width of GB barriers *δ* and further increases the weight of *ρ_b_* in the film resistivity *ρ*. According to the existing piezoresistive model, the PRCs of DRBs (*π_d_*) and GB barriers (*π_δ_*) are lower than that of grain neutral regions *π_g_*. Thus, it implies that the piezoresistive sensitivity of PSNFs with more amorphous contents and smaller grain size should be much lower. However, it is obvious that the deduction is inconsistent with the experiment results. Based on the tunneling piezoresistive theory presented here, the longitudinal PRC of composite GBs *π_bl_* is much larger than that of grain neutral regions *π_gl_* at high doping concentration. As a result of lowering deposition temperature, both the resistivity *ρ_b_* and the weight of *ρ_b_* in the film resistivity *ρ* increase. It enhances the contribution of the PRC *π_bl_* on the piezoresistive sensitivity, thereby increasing longitudinal GFs. For DC PSNFs, the XRD analysis indicates that there are more amorphous phases in the 670°C samples than in the 620°C ones, which is likely due to the <110> preferred growth aggravating disordered states of GBs. It makes the resistivity *ρ_δ_* of 670°C samples higher than 620°C samples. However, the SEM results show that the grain size of 670°C samples is ∼70nm and much larger than that of 620°C ones. This reduces severely the weight of the resistivity *ρ_b_* in the film resistivity *ρ* and weakens the contribution of the PRC *π_bl_* on the piezoresistive sensitivity. Therefore, the longitudinal GF of 670°C samples with larger grains is much lower than that of 620°C ones.

For the transversal piezoresistive sensitivity, the inset of Figure 13(b) shows that the magnitude of the transversal GF in DC PSNFs increases with lowering deposition temperature, similar to the longitudinal GF dependence; while the magnitude of the transversal GF in RC PSNFs falls off drastically with lowering deposition temperature. Comparing the insets of Figure 13(a) and (b), it can be seen that the longitudinal GF of DC PSNFs is about twice larger than the transversal one. However, the longitudinal and transversal GFs of RC PSNFs do not satisfy the above proportional relation. On the contrary, the transversal GF of RC PSNFs decreases from 1/2 to 1/3 of the longitudinal one with lowering deposition temperature, which might be due to the degradation of transversal piezoresistive effect in amorphous silicon.

It is noteworthy that the stress-induced modulation of surface depletion region width in silicon nanowires [3] is not fit for the explanation of enhanced piezoresistive effect in PSNFs. For silicon nanowires, the surface depletion regions are parallel to the direction of carrier transport, and the change in surface potential barrier caused by stress only influences the conducting channel width of carriers along silicon nanowires. However, for PSNFs, the depletion regions are perpendicular to the direction of carrier transport and the carriers have to traverse them by thermionic emission or tunneling. Moreover, the depletion region width is reduced greatly at high doping concentration and can be neglected. So the tunneling effect of carriers becomes dominant.

Although the inset of Figure 13(a) shows that the longitudinal GFs of 560°C and 580°C samples are slightly higher than those of DC PSNFs, it could not be proven that their sensing performance is better. The reliability and stability have to be also taken into consideration. Therefore, the PRL and RTD of PSNFs were investigated in this paper.

## 4. Piezoresistive linearity

The PRL of PSNFs is one of significant parameters estimating the static characteristics of piezoresistive sensors. In this section, the IV model is presented to describe the disordered states at GBs of PSNFs. Based on this model, the influence of high temperature annealing on amorphous phases at GBs is analyzed. Finally, the relationship between the PRL and deposition temperature is discussed and explained using this model.

### 4.1. Interstitial-vacancy model of grain boundaries

Polysilicon is a monatomic silicon material with complex structure. The GBs have a significant impact on the material nature. Particularly, for PSNFs, both the film thickness and the grain size are nanometer sized, so the influence of GBs is enhanced greatly. Furthermore, there are higher amorphous contents existing at GBs of PSNFs, due to insufficient crystallization and fine grains. Thus, the nature of GBs is considered to be amorphous in this paper. In order to describe the amorphous phases at GBs, it is necessary to establish a theoretical model.

According to the work by Marques *et al.* [33], the disordered zones in amorphous silicon can be characterized as the accumulation of interstitial silicon atoms and vacancies. For simplification, these disordered zones are considered as comprised of basic point defects. The point defect structure consists of an interstitial atom and a vacancy and is called interstitial-vacancy pair (IV pair). Similarly, GBs of PSNFs can be modeled by the accumulation of IV pairs.

In the IV model, the amorphous regions at GBs can be classified into continuous amorphous layers (continuous a-layers) and amorphous pockets (a-pockets). And continuous a-layers and a-pockets exhibit similar features. In amorphous regions, the state of each IV pair is dependent on the number of neighboring IV pairs (defined as the IV pair coordination number). Under external energy excitation (e.g., heating, plastic deformation, ion bombardment), an IV pair can overcome the energy barrier of recombination and recombine at a certain probability, similar to the recrystallization of amorphous materials. And the recombination rate increases when the number of neighboring IV pairs decreases. According to the molecular dynamics (MD) calculations by Marques *et al.* [33], the activation energy of the recombination of an isolated IV pair is 0.43eV, while the IV pairs within amorphous regions (surrounded completely by neighboring IV pairs, hence with the full IV pair coordination number) have the activation energy of 5eV. Moreover, it should be noted that the IV pairs located at a planar amorphous/crystal (a/c) interface (with about half of the full coordination number) have the activation energy of 2.7eV [34]. It indicates that the activation energy of IV pair recombination and the number of neighboring IV pairs satisfy the linear interpolation relationship approximately. Thus, the distribution of neighboring IV pairs determines the recombination rate and activation energy of an IV pair.

For continuous a-layers, all the IV pairs at an a/c interface have the same number of neighboring IV pairs, and therefore the same recombination rate. Because the IV pairs at an a/c interface have fewer neighboring IV pairs than in inner zones, the recombination of IV pairs takes place at the a/c interface firstly. It starts a cascade mechanism in which IV pairs could recombine layer by layer. A-pockets show a similar recombination behavior [35]. However, due to their irregular and convex shapes, the IV pairs at the surface of a-pockets have different IV coordination numbers (fewer than those of continuous a-layers), so that the recombination active energies are different. The experiments indicate that the recombination of IV pairs in a-pockets is much faster than that of continuous a-layers [36].

### 4.2. Influence of high temperature annealing on grain boundaries

The original RC PSNFs exhibit amorphous appearance mixed with polysilicon. Here, it is considered that the amorphous contents include continuous a-layers and a-pockets. Therefore, the pre-annealing was performed at 950°C for 30 min to induce the recrystallization of amorphous contents. However, even after the post-implantation annealing at 1080°C, the SEM and XRD results show that there are still many amorphous contents existing at GBs. It indicates that the further recrystallization of amorphous contents was prevented in the temperature range of 950∼1080°C. This is likely related to the concentration of IV pairs at GBs.

The MD simulation results by Marques *et al.* [33] show that when the temperature is around 1200 K, IV pairs with a concentration of 25% in amorphous regions produce polycrystalline material; when the temperature is higher than 1200 K and even 1600 K, IV pairs with a concentration of 25% generate amorphous matrix again. For IV pairs with a concentration of 10-20%, the recrystallization takes place in the temperature range between 1000 and 2000 K. For RC PSNFs, the experiments indicate that the pre-annealing of 950°C (≈1220 K) gives rise to the recrystallization of amorphous contents at GBs and the increase of polysilicon contents, but the post-implantation annealing of 1080°C (≈1350 K) increases amorphous contents to a certain extent. Consequently, the concentration of IV pairs at GBs of RC PSNFs is estimated to be about 25%. For PSCFs, the experimental results show that the annealing of 1080°C can increase the grain size and improve the film crystallinity [15]. It can be considered that GBs of PSCFs contain IV pairs with a concentration of 10-20%. This verifies that the crystallinity of RC PSNFs is worse than that of PSCFs and there are more amorphous phases at GBs of RC PSNFs.

**Figure 14.**
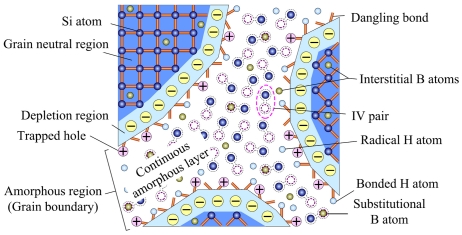
Interstitial-vacancy model of grain boundaries in PSNFs.

In this paper, a-pockets with fewer surface IV coordination numbers are considered to be eliminated during the pre-annealing, due to their faster recrystallization rate. Therefore, GBs of PSNFs can be modeled as continuous a-layers, as shown in Figure 14. Boron dopants are ion-implanted into PSNFs, forming the interstitial atoms and substitutional atoms. The occupation of substitutional boron atoms to the vacancies also suppresses the recrystallization of IV pairs.

### 4.3. Piezoresistive nonlinearity versus deposition temperature

**Figure 15.**
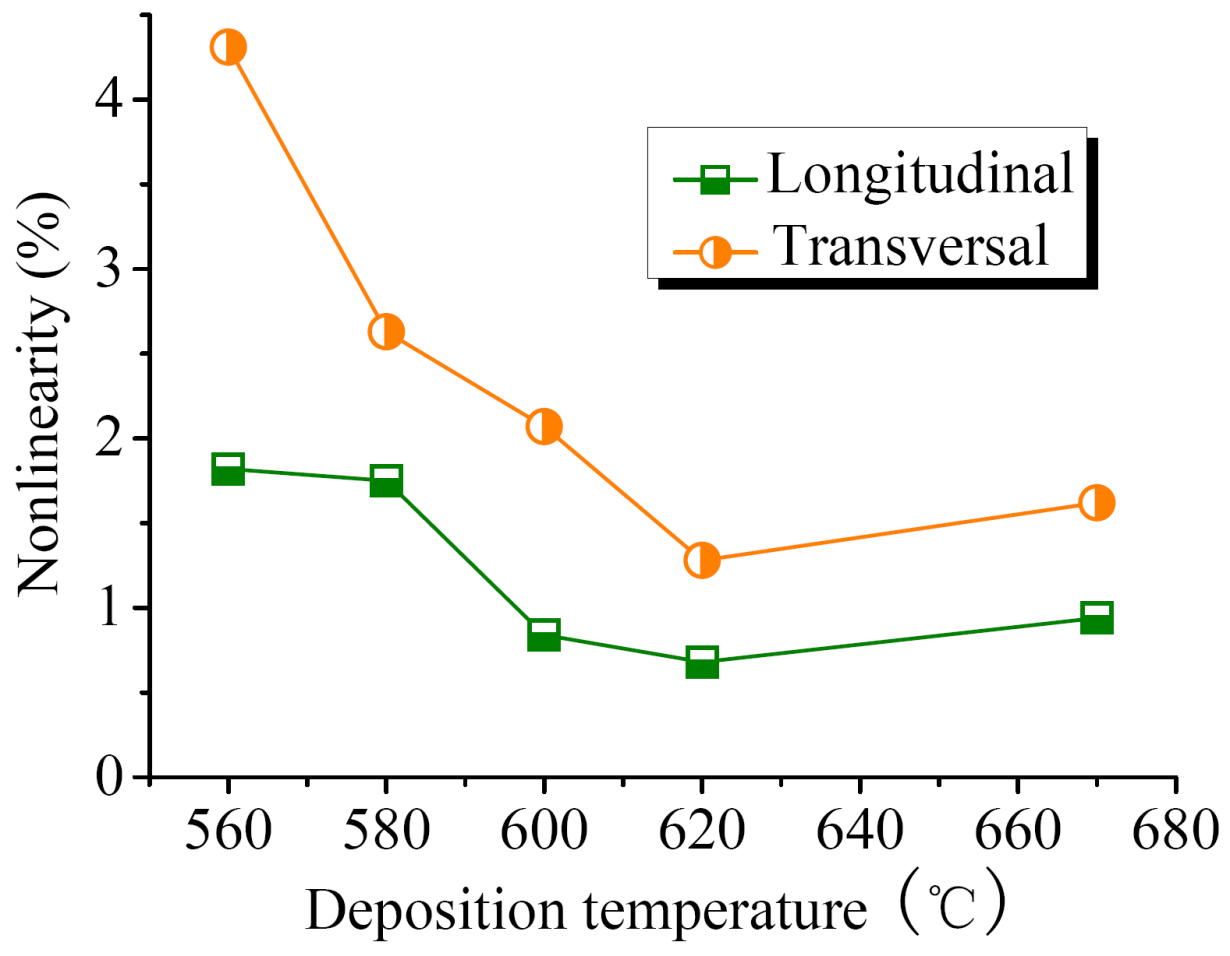
PRNL of longitudinal and transversal PSNF piezoresistors as a function of deposition temperature.

The dependences of PRNL of longitudinal and transversal PSNF piezoresistors on deposition temperature are given in Figure 15. Obviously, the PRL of longitudinal piezoresistors is superior to the transversal ones. It should be noted that the PRNL of RC PSNFs and 670°C samples is higher than 620°C samples, indicating that amorphous phases at GBs are the origin of PRNL. In the case of room temperature and small stresses, only the first-order PRCs are taken into account, and the nonlinear piezoresistance effects (high-order PRCs) in silicon [37] could be neglected. Therefore, the conditions of GBs make a significant impact on the PRL.

In the IV model, GBs are considered to be the accumulation of IV pairs and behave as continuous a-layers. According to the simulation results by Marques *et al.* [33], the IV pair is not stable and the lifetime of an isolated IV pair is only 3μs at room temperature. Even though an isolated IV pair is unstable (the energy barrier of recombination is only 0.43eV), the accumulation of IV pairs could increase the active energy to 5eV (in amorphous matrix) and improve the stability of IV pairs. However, there are still unstable IV pairs existing at GBs, they could recombine under external energy excitation. Due to the existence of GBs as a metastable non-crystal structure, polysilicon is not an absolute rigid material. The slight plastic deformation could occur, when a stress is applied to the material. Similar to the crystallization induced by deformations in non-crystal metals [38], the deformation could increase the energies of inner atoms at GBs and cause some unstable IV pairs (with low energy barrier of recrystallization) to recombine. The recombination of IV pairs caused by material deformation leads the number of scattering centers to change, thereby resulting in the minute variation of film resistivity. This might be one of the prime reasons for PRNL. It should be noted that the change in resistivity caused by the recrystallization of IV pairs is very little and can not influence the change trend of resistance for piezoresistive effect.

Considering the type of applied stresses, the longitudinal piezoresistive effect is caused by tension stress, while the transversal piezoresistive effect is caused by compression stress. The experiment results based on metal materials show that the compression stress has a more remarkable role in stress-induced crystallization than tension stress. Similarly, it is believed that the recombination of IV pairs under compression stress is more prominent. Consequently, the PRNL of transversal piezoresistors is higher than that of longitudinal ones, which is in good agreement with the experimental results in Figure 15. On the other hand, based on the explanations presented in our previous research work [39], the higher amorphous contents increase trap density at GBs, resulting in that the probability of traps capturing heavy and light holes could change. This might be the secondary reason for PRNL.

Finally, the experimental results indicate that the 620°C PSNFs with the lowest amorphous contents have the best PRL. Although the high temperature annealing could improve the crystallinity of RC PSNFs, a large number of residual amorphous contents at GBs still produce the higher PRNL. It testifies that the disordered states of GBs determine the PRL of PSNFs.

## 5. Resistance time drift properties

In this section, the RTD properties of PSNF resistances are investigated. The recombination of unstable IV pairs at GBs and the motion of residual hydrogen atoms are both taken into consideration.

### 5.1. Resistance time drift versus deposition temperature

**Figure 16.**
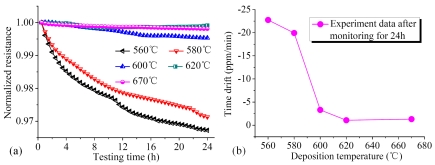
(a) Normalized resistances of PSNF piezoresistors with different deposition temperatures as a function of testing time during monitoring for 24 h. (b) RTD of PSNF resistors versus deposition temperature after monitoring for 24 h.

The RTD is one of the important parameters characterizing the electrical reliability of resistive materials. Figure 16(a) shows the relationship between normalized resistances of PSNF resistors and testing time. It can be seen that the resistances decrease with testing time and the resistance reduction is nonreversible. Moreover, the drift rate of PSNF resistance decreases gradually. It indicates that there exists an ultimate equilibrium for the RTD. The relationship between RTD of PSNF resistors and deposition temperature is shown in Figure 16(b). From this figure, it can be seen that the RTDs of RC and 670°C PSNFs are larger than that of 620°C samples. Similar to PRL, amorphous phases at GBs also play a significant role on the RTD properties. Based on the IV model, under the low field current excitation, the energies of minority unstable IV pairs at GBs increase gradually. When these IV pairs have enough energy to overcome the weak energy barrier of recombination, the IV pair recrystallization could take place. The recrystallization also reduces the number of scattering centers, thereby resulting in the decrease of PSNF resistance with testing time. With the number of unstable IV pairs decreasing, the drift rates of PSNF resistances decrease gradually and drive to the ultimate equilibrium.

### 5.2. Influence of residual hydrogen atoms at grain boundaries

The experiments evidenced that CVD polysilicon films contain residual hydrogen atoms in bonded or radical forms from the processing ambient. According to the constrained dynamics, the mobile hydrogen atoms are difficult to occur in the crystallized silicon, and most of hydrogen atoms are distributed into GBs, as shown in Figure 14. Usually, these hydrogen atoms are bonded with silicon dangling bonds at GBs, or passivate the boron dopant atoms. However, due to the weak strength of Si-H bond (3.55eV) [40] and B-H bond (4.31eV) [41], a small fraction of bonded hydrogen atoms might escape from the original bonding Si or B atoms, forming radical hydrogen atoms. Under external current excitation, these radical hydrogen atoms can be captured by dangling bonds or defects at GBs. The occupation of hydrogen atoms to these sites gives rise to the motion of hydrogen atoms at GBs, which reduces the number of dangling bonds and scattering centers, thereby resulting in the decrease of PNSF resistance. Obviously, the reaction between radical hydrogen atoms and these active sites could drive to the ultimate equilibrium, so that the drift rates of resistances decrease gradually. Thus, it is necessary to passivate these dangling bonds and defects with stronger covalent bonds (e.g., Si-F bond [42], Si-S bond [43], etc.) to improve the electrical stability of PSNFs.

Figure 16(b) illustrates that the PSNFs deposited at 620°C possess the lowest RTD value. It indicates that reducing dangling bonds and defects at GBs by optimizing deposition temperature could improve the long-term stability of PSNFs. Nevertheless, the recrystallization after high temperature annealing can not suppress the RTD effectively. It is due to that the high concentration (∼25%) of IV pairs at GBs in RC PSNFs impedes the transfer from amorphous phases to polycrystalline phases. Finally, it is achieved that the optimal deposition temperature for reliable and stable PSNFs is around 620°C.

## 6. Conclusions

In this paper, the piezoresistive sensitivity, PRL and RTD of highly boron doped LPCVD PSNFs were studied, and the influences of deposition temperature and high temperature annealing on the above-mentioned properties were analyzed. As for the tunneling piezoresistive effect, the tunneling piezoresistive model was established. The carrier transport mechanisms across GBs are considered to be thermionic emission and tunneling, and the tunneling current becomes dominant at high doping concentration. By theoretical calculation, a conclusion was drawn that the PRC of composite GBs is higher than that of grain neutral regions at high doping concentration. The dependences of longitudinal and transversal GFs on deposition temperature were obtained. The results indicate that the piezoresistive properties of the RC PSNFs are different from the DC ones. According to SEM and XRD results, it is considered that amorphous contents at GBs and grain size are important factors influencing the piezoresistive properties of PSNFs. The magnitude of GF is dependent on the equivalent resistivity of GB barriers and the weight of the resistivity of composite GBs in the film resistivity.

In the investigations on PRL and RTD of PSNF piezoresistors, the IV model was established to describe the nature of GBs. In our model, GBs are regarded as the accumulation of IV pairs. The PRNL is due to the reduction in the number of scattering centers caused by the stress-induced recrystallization of metastable IV pairs at GBs. For RTD of PSNFs, it is considered as due to the recombination of minority IV pairs under low field current excitation. And the motion of residual hydrogen atoms at GBs is also taken into account. Consequently, the conclusions were drawn that the deposition temperature is one of the significant parameters determining the nature of PSNFs. Optimizing the structure of grains and GBs by controlling deposition temperature could improve the piezoresistive performance of PSNFs. Even though the high temperature annealing could induce the recrystallization of amorphous contents at GBs, the transfer from amorphous phases to polycrystalline phases is dependent on the concentration of IV pairs at GBs and annealing temperature. The high temperature annealing on the PSNFs with high concentration (∼25%) of IV pairs is not very effective in improving the reliability and stability of the material. Finally, it is obtained that the optimal deposition temperature is about 620°C.

## Figures and Tables

**Table 1.** Hole effective mass in highly stressed silicon (unit: free-electron mass m_0_).

*m*_l1_	*m*_t1_	*m*_d1_	*m*_l2_	*m*_t2_	*m*_d2_
0.870	0.170	0.293	0.135	0.369	0.264
